# Editorial: Molecular dynamics of cognitive-motor impairment in neurodegenerative diseases

**DOI:** 10.3389/fnmol.2023.1237769

**Published:** 2023-06-22

**Authors:** Dhiraj Kumar, Saba Noor, Manzar Alam, Md Imtaiyaz Hassan

**Affiliations:** ^1^Section of Retinal Ganglion Cell Biology, Laboratory of Retinal Cell and Molecular Biology, National Eye Institute, National Institutes of Health, Bethesda, MD, United States; ^2^Centre for Interdisciplinary Research in Basic Sciences, Jamia Millia Islamia, New Delhi, India; ^3^Department of Pathology, UT Southwestern Medical Center, Dallas, TX, United States

**Keywords:** motor, cognition, misfolded proteins, protein dynamics, neurodegenerative diseases

A substantial percentage of neuroscience research has focused on decision-making and guided actions. Impaired cognition and motor dysfunction are typical hallmarks of several neurodegenerative disorders, but the type of these abnormalities varies by disease (Kumar et al., 2020). Examining the qualitative and quantitative differences in cognitive-motor impairments associated with different neurodegenerative diseases could thus provide useful information about the underlying neurological foundation of human behavior and action. In this Research Topic, we look at the neuroanatomical bases of cognitive and motor functions to the neuropathological changes in different neurodegenerative diseases and traumatic brain injury. Here, we looked at the recent key players governing the dynamics of cognitive-motor function and made recommendations for future research to improve our understanding of cognitive behavior and motor dysfunctions in neurodegenerative diseases.

## Neuroanatomical bases of cognitive and motor dysfunctions

Several factors have been reported, including abnormal protein dynamics, accumulation of misfolded proteins, ubiquitin–proteosome–autophagy system alterations, and impaired bioenergetics. Recent findings unfolded many aspects of brain cognition, emotion, and motor functions in human biology.

### Septin genes

Several genes, such as Septin-14, are particularly prevalent in the embryonic and newborn brain, signaling an essential function in guiding, structuring, and pruning the early-life brain's developing neuronal circuits and modules. Chen et al. revealed that in both sexes of adult mice, deletion of the Septin-14 gene enhances ventral hippocampus DG cell proliferation and stress-induced anxiety while decreasing observational fear conditioning magnitudes. While multiple septin GTPases have been found for physiological purposes, less emphasis has been paid to their roles in coordinating advanced cognitive/emotional functions in mature animals. The sexually dimorphic effects of brain SEPT14 KO on inhibitory avoidance (IA) and hippocampal mGluR5 expression were only seen in male KO mice, who had longer IA latency and higher mGluR5 levels. Furthermore, independent of the animals' sexes, SEPT14 KO was linked with increased stress-induced anxiety in a stress-related navigation task. While male and female WT mice showed similar cell proliferation in the dorsal and ventral hippocampal dentate gyrus (DG), SEPT14 KO mice had increased cell proliferation in the ventral DG in both sexes (Chen et al.). Overall, these findings suggest that male, but not female, mice lacking the Septin-14 gene have higher unpleasant emotion-related learning and dorsal/ventral hippocampus mGluR5 expression, which can be linked to increased ventral hippocampus DG cell proliferation and stress-induced anxiety-like behavior, while decreasing vicarious fear conditioning magnitudes.

### Alpha-synuclein

Growing evidence shows that aberrant α-syn accumulation in the hippocampus might lead to cognitive impairment. Overexpression of α-syn in nuclei was shown by Pan et al. to induce considerable pathological accumulation of α-syn in the hippocampus, leading to memory and motor deficits in mice. Nuclear overexpression of α-syn is thought to cause DNA damage in hippocampus neurons, resulting in activation, aberrant cell cycle blockage, hippocampal neuron death, and inflammatory response. Meanwhile, the inflammatory response exacerbated DNA damage and created a vicious cycle, resulting in Parkinson's disease symptoms (Pan et al.).

### Obesity

Obesity has become a pandemic in recent decades and is often regarded as one of the world's most serious public health issues. Obesity caused by a high-fat diet (HFD) significantly contributes to decreased memory and cognitive function, but the underlying processes remain unknown. The current study examined the amounts of circRNAs in the hippocampus of C57BL/6J mice and the memory and cognitive abilities of C57BL/6J mice fed HFD utilizing the Morris water maze and Y-maze methods. HFD-induced obesity mice with variable circRNA expression (mmu-circRNA-004797 and mmu-circRNA-21040) demonstrated decreased synaptic plasticity and endoplasmic reticulum stress in hippocampus neurons with obesity-associated cognitive impairment (Niu et al.). This study revealed that circRNAs play crucial functions and are most likely implicated in obesity-related cognitive impairment susceptibility.

### Hand grip strength

Interestingly, Huang et al. evaluated the relationship between hand grip strength and cognitive decline in elderly Americans. On 2,623 people aged 60 years, the Consortium to Establish a Registry for Alzheimer's Disease (CERAD < 5), animal fluency (AF < 14), and digit symbol substitution test (DSST < 34) scores were used to assess cognitive impairment (Huang et al.). They discovered an inverse link between hand grip strength and cognitive impairment, which might point to a common underlying mechanism that should be studied further in large-scale prospective clinical studies.

### Traumatic brain injury

TBI is one of the top causes of death and disability worldwide, with a complex and variable process. Glutamate-induced excitotoxicity, oxidative stress, inflammatory response, ion imbalance, and metabolic disarray, for example, are all important pathogenic alterations that lead to neuronal death (Rosenfeld et al., 2012). While high calcium influx causes mitochondrial disintegration, increased BBB permeability and lactic acidosis create cerebral edema, intracranial hypertension causes distinct pathologic alterations (Maas et al., 2017). Here, neurological abnormalities exist from the acute to the subacute stages, and brain parenchyma was significantly affected at either stage. The dynamic pathophysiology of these stages revealed candidate targets ranging from platelet activation to nerve-related pathways to some aberrant secretions that were crucial in the acute phase. The considerable enrichment of amyotrophic lateral sclerosis, on the other hand, alerts us to the continuation of nerve damage into the subacute phase, implying that neuroprotective intervention should be implemented at the earliest stage of severe traumatic brain injury to minimize cognitive motor dysfunction (Luo et al.).

## Recommendations for future research

There are few motor-cognition comparison reports on the pathological changes among different neurodegenerative diseases. Raghav et al. provided an extensive review of the potential of extracellular vesicles in improving cognitive-motor functions by curbing neuro-inflammation. Both indications are frequently seen in the same condition, making distinguishing “pure” motor or cognitive diseases challenging. Movement issues are typical in neurodegenerative dementias, and cognitive deficits frequently accompany them. Such phenotypic overlaps advise treating these problems by emphasizing the similarities between organisms historically deemed different (Kumar et al., 2022). Further, emerging technologies of extracellular vesicle packaging with targeted short RNAs can guide the future therapeutics of cognitive-motor dysfunction in neurodegenerative diseases.

## Author contributions

This Research Topic on *Molecular dynamics of cognitive-motor impairment in neurodegenerative diseases* was originally conceived and set up by DK. All of the editors collaborated to select which articles were accepted or rejected and the panel of editors and peer reviewers reviewed each manuscript. DK and MH oversaw this editorial introduction. However, the editing team provided ideas and modifications to help shape the final document. All authors contributed to the article and approved the submitted version.
